# HIV-1 disease progression is associated with bile-salt stimulated lipase (BSSL) gene polymorphism

**DOI:** 10.1186/1742-4690-8-S2-P65

**Published:** 2011-10-03

**Authors:** Martijn J Stax, Neeltje A Kootstra, Angélique B van't Wout, Margreet Bakker, Georgios Pollakis, William A Paxton

**Affiliations:** 1 Lab of Exp Virology, Academic Medical Center of the Univ of Amsterdam (AMC; 2 Dept Exp Immun, Sanquin Res, Landsteiner Lab, and CINIMA, AMC, Amsterdam, The Netherlands

## Background

Among cell types targeted by HIV-1 are dendritic cells (DCs), which play a central role in protection against infections and activation of anti-microbial immune responses. HIV-1 binds pathogen receptor DC-SIGN expressed by DCs and is transferred to CD4^+^ T-lymphocytes. We previously identified BSSL (bile-salt stimulated lipase) as the DC-SIGN binding and HIV-1 transfer blocking glycoprotein in human milk. *In vitro* DC-SIGN binding properties of human milk associated with a variation in the number of repeats encoded by exon 11 of the BSSL gene. BSSL is not only expressed in milk but also in blood and blood BSSL binds to CXCR4. We hypothesized that BSSL in blood interferes with the interaction of CXCR4 and DC-SIGN with HIV-1 and thus influences HIV-1 infection and HIV-1 disease progression.

## Materials and methods

The relation between BSSL exon 11 repeat numbers and HIV-1 transmission and disease course as well as the emergence of CXCR4-using variants were studied using Kaplan Meier and multivariate Cox proportional hazard analysis. Additionally, the association of BSSL genotype with CD4 cell count was analyzed, both pre-infection and post-infection at viral setpoint. The study group existed of a cohort of men having sex with men and included 334 seropositive and 48 high risk seronegative individuals (HRSN) participating in the Amsterdam Cohort Studies on HIV infection and AIDS.

## Results

The number of repeats in BSSL exon 11 were highly variable ranging from 10 to 18 in seropositive individuals and from 5-17 in HRSN with 16 repeats being dominant (82% in the seropositive and 85% in the HRSN group carry at least one allele with 16 repeats). As compared to seropositives, a trend was observed with HRSN having more frequently two alleles with 16 repeats (p=0.059). We then defined 16 to 18 repeats as high (H) and less than 16 repeats as low (L) repeat numbers. Individuals have either two low (LL), one low and one high (LH) or two high repeat number alleles (HH). The HH BSSL genotype associated with high CD4 cell numbers prior to HIV-1 infection (p=0.007). No differences were found between BSSL genotypes in CD4 count and virus load at viral setpoint post seroconversion. However, the HH BSSL genotype was linked to slow disease progression (p=0.049, RH=0.737) and delayed emergence of CXCR4-using HIV-1 variants (p=0.007, RH=0.515).

## Conclusion

We identified BSSL as a marker for HIV-1 disease progression, CXCR4 using HIV-1 variant emergence and CD4 cell homeostasis. Knowledge on the identified BSSL genotypes may help develop a new class of therapeutic molecules aimed at treating HIV-1 disease. (Dutch AIDS fund grant 2005024)

